# Supplementary material for: P-TRAP: a Panicle Trait Phenotyping tool
Source: BMC Plant Biol. 2013 Aug 29;13:122. doi: 10.1186/1471-2229-13-122 (PMC3848748; doi:10.1186/1471-2229-13-122)
Supplement: Additional file 7 — P-TRAP source code. The Netbeans project that contains the source code of the application. [file 1471-2229-13-122-S7.zip › javadoc.zip!/javadoc/org-bioalg-imageprocessor/index-all.html]

Index (Image Processor)


JavaScript is disabled on your browser.


- Package
- Class
- Tree
- Deprecated
- Index
- Help

*org.bioalg.imageprocessor 1.0*

- Prev
- Next

- Frames
- No Frames

- All Classes

A B C D E F G H I K L M N O P R S T V W Z 


## A

AImage - Class in org.bioalg.imageprocessor
:   The main class used for image processing of the project.

AImage(int, int, int) - Constructor for class org.bioalg.imageprocessor.AImage


AImage(AImage) - Constructor for class org.bioalg.imageprocessor.AImage
:   Copy constructor

AImage(int[][], int) - Constructor for class org.bioalg.imageprocessor.AImage
:   From int matrix with a given bitdepth

AImage(double[][], int) - Constructor for class org.bioalg.imageprocessor.AImage
:   From a double matrix with a given bitDepth

AImage.CONNECTIVITY - Enum in org.bioalg.imageprocessor


AND(AImage) - Method in class org.bioalg.imageprocessor.AImage
:   The logical ANDing process between this image and the input image.


## B

bilinearInterp(double, double) - Method in class org.bioalg.imageprocessor.AImage
:   The bilinear interpolation of two values


## C

close(int[][]) - Method in class org.bioalg.imageprocessor.MathMorphology
:   The closing task.

ColoredImage - Class in org.bioalg.imageprocessor
:   The colored image class.

ColoredImage(int, int) - Constructor for class org.bioalg.imageprocessor.ColoredImage
:   An empty image constructor.

ColoredImage(BufferedImage) - Constructor for class org.bioalg.imageprocessor.ColoredImage
:   Create a colored image from a bufferedImage.

convertType(File, File, String) - Static method in class org.bioalg.imageprocessor.AImage
:   Convert between image types.

convertType(File, File, String) - Static method in class org.bioalg.imageprocessor.ImageIO
:   Convert image types

convolve2d(double[][]) - Method in class org.bioalg.imageprocessor.AImage
:   The 2D convolution of the current image.

createImage(int[][], int, int) - Static method in class org.bioalg.imageprocessor.ImageIO
:   Create an image given the data in an integer matrix.

createImageDouble(double[][], int, int) - Static method in class org.bioalg.imageprocessor.ImageIO
:   Create a double-valued image.


## D

denormalize() - Method in class org.bioalg.imageprocessor.AImage
:   Restores the normalized image from [0-1] to [0-maxInt]

dilate(int[][]) - Method in class org.bioalg.imageprocessor.MathMorphology
:   Dilate a binary image by using a structure element.

distance(HSBPixel) - Method in class org.bioalg.imageprocessor.HSBPixel


distance(RGBPixel) - Method in class org.bioalg.imageprocessor.RGBPixel


distancehs(HSBPixel) - Method in class org.bioalg.imageprocessor.HSBPixel


distances(HSBPixel) - Method in class org.bioalg.imageprocessor.HSBPixel


distancesb(HSBPixel) - Method in class org.bioalg.imageprocessor.HSBPixel


## E

EDistanceMap - Class in org.bioalg.imageprocessor
:   The distance mapper of the image.

EDistanceMap() - Constructor for class org.bioalg.imageprocessor.EDistanceMap


EDT\_1D(AImage) - Static method in class org.bioalg.imageprocessor.EDistanceMap
:   Perform the first step of 1D Distance mapping

equals(Object) - Method in class org.bioalg.imageprocessor.RGBPixel


erode(int[][]) - Method in class org.bioalg.imageprocessor.MathMorphology
:   Eroding the image by a structure element.

erode1(int[][]) - Method in class org.bioalg.imageprocessor.MathMorphology
:   to be reworked


## F

fastMeanThresholding(int, double) - Method in class org.bioalg.imageprocessor.AImage
:   An optimized version of the mean local threshold method

fillholes(int) - Method in class org.bioalg.imageprocessor.AImage
:   A fill-holes algorithm based on finding a suitable seed on the
    background, fill the background by a color then replace this color in the
    filled image by backgorund and replace the rest by foreground color!

fillsmallholes(int, int) - Method in class org.bioalg.imageprocessor.AImage
:   Small holes filling algorithm, it is based on some mathematical basic
    operations and the holes filling classical approach

findconnectedcompnents(AImage, int) - Method in class org.bioalg.imageprocessor.AImage
:   Find the connected components in the image.

floodfill(Point, Point, int, int) - Method in class org.bioalg.imageprocessor.AImage
:   Implements the breadth-first flood fill algorithm

floodfill2(Point, Point, int, int) - Method in class org.bioalg.imageprocessor.AImage
:   Implements the depth-first flood fill algorithm


## G

gaussianBlur(double) - Method in class org.bioalg.imageprocessor.AImage
:   The Gaussian blurring.

generateCross(int) - Static method in class org.bioalg.imageprocessor.Kernels


generatedagger(int) - Static method in class org.bioalg.imageprocessor.Kernels


generateDiskAsPoints(int, Point) - Static method in class org.bioalg.imageprocessor.Kernels
:   generate a disk mask for math morphology

generateDiskKernel(int) - Static method in class org.bioalg.imageprocessor.Kernels
:   generate a disk mask for math morphology

generateEllipseKernel(int, int) - Static method in class org.bioalg.imageprocessor.Kernels
:   generate an ellipse mask for math morphology

generateemptyDiskKernel(int) - Static method in class org.bioalg.imageprocessor.Kernels


generateframe(int) - Static method in class org.bioalg.imageprocessor.Kernels


generateframe(int, int) - Static method in class org.bioalg.imageprocessor.Kernels


generateframe(int, Point) - Static method in class org.bioalg.imageprocessor.Kernels


generateframe(int, int, Point) - Static method in class org.bioalg.imageprocessor.Kernels


generateInvertedDiskKernel(int) - Static method in class org.bioalg.imageprocessor.Kernels


generateSqureKernel(int) - Static method in class org.bioalg.imageprocessor.Kernels


getB() - Method in class org.bioalg.imageprocessor.HSBPixel


getB() - Method in class org.bioalg.imageprocessor.RGBPixel


getcircularNxNmatrix(int, int, int) - Method in class org.bioalg.imageprocessor.AImage
:   Return a circular matrix centered at row i and column j of the original
    image.

getcircularNxNmatrixROI(int, int, ArrayList<Point>, int) - Method in class org.bioalg.imageprocessor.AImage
:   Return a circular matrix centered at row i and column j of the original
    image.

getconnectedObject(Point, Point, int, int) - Method in class org.bioalg.imageprocessor.AImage
:   Implements the depth-first flood fill algorithm to find a connected
    object

getconnectedObject2(Point, Point, int, int) - Method in class org.bioalg.imageprocessor.AImage


getDepth() - Method in class org.bioalg.imageprocessor.AImage


getEntropy() - Method in class org.bioalg.imageprocessor.AImage
:   Calculate the entropy of the image's histogram.

getFGPixels(ArrayList<Point>) - Method in class org.bioalg.imageprocessor.AImage
:   Return the white pixels in a ROI.

getfirstvector(int, int, int) - Method in class org.bioalg.imageprocessor.AImage
:   Get the first vector of a kernel of nXn kernel

getFullGaussian(double) - Static method in class org.bioalg.imageprocessor.Kernels


getFullLoG(double) - Static method in class org.bioalg.imageprocessor.Kernels


getG() - Method in class org.bioalg.imageprocessor.RGBPixel


getH() - Method in class org.bioalg.imageprocessor.HSBPixel


getHeight() - Method in class org.bioalg.imageprocessor.ColoredImage


getHistogram() - Method in class org.bioalg.imageprocessor.AImage
:   Calculate the histogram of the image.

getHistogramInt() - Method in class org.bioalg.imageprocessor.AImage
:   Return an integer histogram of the image.

getImageAsAImage(PlanarImage) - Static method in class org.bioalg.imageprocessor.ImageIO
:   Convert a `PlanarImage` to a `AImage`.

getImageAsGrayscaledAImage(File) - Static method in class org.bioalg.imageprocessor.ImageIO
:   Load and return a grayscale version of the image

getlastvector(int, int, int) - Method in class org.bioalg.imageprocessor.AImage
:   Return the last vector of a kernel of nXn matrix

getMatrix() - Method in class org.bioalg.imageprocessor.AImage
:   Return the image matrix.

getMatrix() - Method in class org.bioalg.imageprocessor.ColoredImage


getNbyNsubmatrix(int, int, int) - Method in class org.bioalg.imageprocessor.AImage
:   Return an nXn matrix of the image given a pixel position (i,j)

getNormMaxMin() - Method in class org.bioalg.imageprocessor.AImage
:   Returns a normalized image, with intensities scaled to max-min
    intensities.

getNormMaxMin(int) - Method in class org.bioalg.imageprocessor.AImage
:   Get a normalized version of the image scaled from 0 to a bound.

getR() - Method in class org.bioalg.imageprocessor.RGBPixel


getRC(int, int, double) - Method in class org.bioalg.imageprocessor.AImage
:   Get the pixel value at a given row and column.

getRC(int, int, RGBPixel) - Method in class org.bioalg.imageprocessor.ColoredImage
:   Get the pixel at a given row and column.

getS() - Method in class org.bioalg.imageprocessor.HSBPixel


getScaledAImage(double) - Method in class org.bioalg.imageprocessor.AImage
:   Returns a new object of AImage scaled by scaleFactor

getSourceAImage() - Method in class org.bioalg.imageprocessor.MathMorphology


getSubImage(int, int, int, int) - Method in class org.bioalg.imageprocessor.AImage
:   Return a sub image given four points.

getsumOfROI(ArrayList<Point>) - Method in class org.bioalg.imageprocessor.AImage
:   Get the sum of the pixels in a ROI region

getTotalIntensities() - Method in class org.bioalg.imageprocessor.AImage
:   The possible number of intensities according to the bitdepth

getWidth() - Method in class org.bioalg.imageprocessor.ColoredImage


## H

height() - Method in class org.bioalg.imageprocessor.AImage
:   The height of the image

horizontalLinesKernel - Static variable in class org.bioalg.imageprocessor.Kernels


HSBPixel - Class in org.bioalg.imageprocessor
:   The Hue Saturation Brightness model.

HSBPixel(float, float, float) - Constructor for class org.bioalg.imageprocessor.HSBPixel


HSBPixel(RGBPixel) - Constructor for class org.bioalg.imageprocessor.HSBPixel


## I

ImageIO - Class in org.bioalg.imageprocessor
:   The Input Output operations of the image based on both generic java imaging
    and JAI imageing.

ImageIO() - Constructor for class org.bioalg.imageprocessor.ImageIO


invert() - Method in class org.bioalg.imageprocessor.AImage


isclean(int[][]) - Method in class org.bioalg.imageprocessor.AImage
:   Return true if the matrix contains all zeros

isdarkBackGround() - Method in class org.bioalg.imageprocessor.AImage
:   Given a binary image indicate whether it has a dark or white background.


## K

Kernels - Class in org.bioalg.imageprocessor
:   Several kernels created by this class.

Kernels() - Constructor for class org.bioalg.imageprocessor.Kernels


## L

LoadColoredImage(File) - Static method in class org.bioalg.imageprocessor.ImageIO
:   Load a colored image form a file.


## M

MathMorphology - Class in org.bioalg.imageprocessor
:   The Mathematical morphology handler.

MathMorphology() - Constructor for class org.bioalg.imageprocessor.MathMorphology


MathMorphology(AImage) - Constructor for class org.bioalg.imageprocessor.MathMorphology


MathMorphology.MorphingType - Enum in org.bioalg.imageprocessor


max() - Method in class org.bioalg.imageprocessor.AImage


meanLowpass(int) - Method in class org.bioalg.imageprocessor.AImage
:   Fast mean smoothing method.

meanLowpassing(int, double) - Method in class org.bioalg.imageprocessor.AImage
:   A blurring mean filter

meanThresholding(int, double) - Method in class org.bioalg.imageprocessor.AImage
:   Deprecated.

meijster(AImage) - Static method in class org.bioalg.imageprocessor.EDistanceMap
:   The meijster EDM algorithm

meijsterFunction(int, int, double) - Static method in class org.bioalg.imageprocessor.EDistanceMap


myLog(double, double) - Method in class org.bioalg.imageprocessor.AImage


## N

normalize() - Method in class org.bioalg.imageprocessor.AImage
:   Normalize the image with respect to the possible number if intensities.

normalizeHistogram(double[]) - Method in class org.bioalg.imageprocessor.AImage
:   Normalize the histogram and put it between 0 and 1.

normalizeKernel(double[][]) - Static method in class org.bioalg.imageprocessor.Kernels


## O

open(int[][]) - Method in class org.bioalg.imageprocessor.MathMorphology
:   The Opening task.

OR(AImage) - Method in class org.bioalg.imageprocessor.AImage
:   The logical ORing process.

org.bioalg.imageprocessor - package org.bioalg.imageprocessor


outline(int[][]) - Method in class org.bioalg.imageprocessor.MathMorphology
:   Calculate the contour of binary particles by using simple math morphology
    tasks.


## P

printintKernel(int[][]) - Static method in class org.bioalg.imageprocessor.Kernels


printKernel(double[][]) - Static method in class org.bioalg.imageprocessor.Kernels


printMatrix() - Method in class org.bioalg.imageprocessor.AImage


printpointKernel(ArrayList<Point>, int) - Static method in class org.bioalg.imageprocessor.Kernels


putMark(Point, int, int) - Method in class org.bioalg.imageprocessor.AImage
:   Put a given rectangular mark with a given color


## R

reflect(int[][]) - Static method in class org.bioalg.imageprocessor.Kernels
:   used in eroding a binary image using a dilate code

removecolor(int, int) - Method in class org.bioalg.imageprocessor.AImage
:   Replace the pixels of a given color by another color.

removeComponent(ArrayList<Point>) - Method in class org.bioalg.imageprocessor.AImage
:   Remove a given connected component.

RGBPixel - Class in org.bioalg.imageprocessor
:   The Red Green Blue model of the pixel.

RGBPixel() - Constructor for class org.bioalg.imageprocessor.RGBPixel


RGBPixel(int, int, int) - Constructor for class org.bioalg.imageprocessor.RGBPixel


RGBPixel(int) - Constructor for class org.bioalg.imageprocessor.RGBPixel


## S

sauvolaThresholding(int, double) - Method in class org.bioalg.imageprocessor.AImage
:   The Sauvola's local thresholding

saveAImageAsImage(AImage, String) - Static method in class org.bioalg.imageprocessor.ImageIO
:   Store a `AImage` to a file.

saveAImageAsImage(AImage, String, String) - Static method in class org.bioalg.imageprocessor.ImageIO
:   Store the input image to a file with a given type.

saveAImageAsImageFloat(AImage, String) - Static method in class org.bioalg.imageprocessor.ImageIO
:   Store the input image as floating point.

setB(float) - Method in class org.bioalg.imageprocessor.HSBPixel


setB(int) - Method in class org.bioalg.imageprocessor.RGBPixel


setbyThreshold(int, int, double) - Method in class org.bioalg.imageprocessor.AImage
:   Threshold a given pixel by a threshold

setDepth(int) - Method in class org.bioalg.imageprocessor.AImage


setG(int) - Method in class org.bioalg.imageprocessor.RGBPixel


setH(float) - Method in class org.bioalg.imageprocessor.HSBPixel


setMatrix(RGBPixel[][]) - Method in class org.bioalg.imageprocessor.ColoredImage


setR(int) - Method in class org.bioalg.imageprocessor.RGBPixel


setRC(int, int, double, String) - Method in class org.bioalg.imageprocessor.AImage
:   Set the pixel's value at row r and column to another value val.

setRC(int, int, RGBPixel) - Method in class org.bioalg.imageprocessor.ColoredImage
:   Sets the pixel at the row r and column c by another pixel.

setS(float) - Method in class org.bioalg.imageprocessor.HSBPixel


setSourceAImage(AImage) - Method in class org.bioalg.imageprocessor.MathMorphology


sharpen() - Static method in class org.bioalg.imageprocessor.Kernels


simpleSegmentation(RGBPixel, RGBPixel) - Method in class org.bioalg.imageprocessor.ColoredImage
:   Performs a simple color thresholding by classifying the pixels based on
    the distance to the background and object the as background (0) or object
    (255) by measuring

simpleSegmentation(HSBPixel, HSBPixel) - Method in class org.bioalg.imageprocessor.ColoredImage
:   Performs a simple color thresholding by classifying the pixels based on
    the distance to the background and object the as background (0) or object
    (255) by measuring

smoothMedianNxN(int) - Method in class org.bioalg.imageprocessor.AImage
:   The median filter.

subtract(AImage) - Method in class org.bioalg.imageprocessor.AImage
:   Subtract and image form this image


## T

toBinary(double) - Method in class org.bioalg.imageprocessor.AImage
:   Simple global threshold segmentation method

toInteger() - Method in class org.bioalg.imageprocessor.RGBPixel


toString() - Method in class org.bioalg.imageprocessor.HSBPixel


toString() - Method in class org.bioalg.imageprocessor.RGBPixel


## V

validatepixels(ArrayList<Point>) - Method in class org.bioalg.imageprocessor.AImage
:   To calcuate how many pixels in the "points" param correspond to white
    pixels.

validatePixels(int[][]) - Method in class org.bioalg.imageprocessor.AImage
:   The same as {

valueOf(String) - Static method in enum org.bioalg.imageprocessor.AImage.CONNECTIVITY
:   Returns the enum constant of this type with the specified name.

valueOf(String) - Static method in enum org.bioalg.imageprocessor.MathMorphology.MorphingType
:   Returns the enum constant of this type with the specified name.

values() - Static method in enum org.bioalg.imageprocessor.AImage.CONNECTIVITY
:   Returns an array containing the constants of this enum type, in
    the order they are declared.

values() - Static method in enum org.bioalg.imageprocessor.MathMorphology.MorphingType
:   Returns an array containing the constants of this enum type, in
    the order they are declared.

verticalLinesKernel - Static variable in class org.bioalg.imageprocessor.Kernels


## W

width() - Method in class org.bioalg.imageprocessor.AImage
:   The width of the image


## Z

zeroOneImage() - Method in class org.bioalg.imageprocessor.AImage
:   Convert a binary image to a zero-one image.

A B C D E F G H I K L M N O P R S T V W Z

- Package
- Class
- Tree
- Deprecated
- Index
- Help

*org.bioalg.imageprocessor 1.0*

- Prev
- Next

- Frames
- No Frames

- All Classes
